# Estimating HIV incidence among key affected populations in China from serial cross-sectional surveys in 2010–2014

**DOI:** 10.7448/IAS.19.1.20609

**Published:** 2016-03-16

**Authors:** Yan Cui, Wei Guo, Dongmin Li, Liyan Wang, Cynthia X Shi, Ron Brookmeyer, Roger Detels, Lin Ge, Zhengwei Ding, Zunyou Wu

**Affiliations:** 1 Division of Epidemiology, National Center for AIDS/STD Control and Prevention, Chinese Center for Disease Control and Prevention, Beijing, China; 2 Division of Prevention and Intervention, National Center for AIDS/STD Control and Prevention, Chinese Center for Disease Control and Prevention, Beijing, China; 3 Department of Biostatistics, UCLA Fielding School of Public Health, Los Angeles, CA, USA; 4 Department of Epidemiology, UCLA Fielding School of Public Health, Los Angeles, CA, USA; 5 National Center for AIDS/STD Control and Prevention, Chinese Center for Disease Control and Prevention, Beijing, China

**Keywords:** HIV, cross-sectional survey, incidence, prevalence, mortality, key affected populations

## Abstract

**Introduction:**

HIV incidence is an important measure for monitoring the development of the epidemic, but it is difficult to ascertain. We combined serial HIV prevalence and mortality data to estimate HIV incidence among key affected populations (KAPs) in China.

**Methods:**

Serial cross-sectional surveys were conducted among KAPs from 2010 to 2014. Trends in HIV prevalence were assessed by the Cochran-Armitage test, adjusted by risk group. HIV incidence was estimated from a mathematical model that describes the relationship between changes in HIV incidence with HIV prevalence and mortality.

**Results:**

The crude HIV prevalence for the survey samples remained stable at 1.1 to 1.2% from 2010 to 2014. Among drug users (DUs), HIV prevalence declined from 4.48 to 3.29% (*p*<0.0001), and among men who have sex with men (MSM), HIV prevalence increased from 5.73 to 7.75% (*p*<0.0001). Changes in HIV prevalence among female sex workers (FSWs) and male patients of sexually transmitted disease clinics were more modest but remained statistically significant (all *p*<0.0001). The MSM population had the highest incidence estimates at 0.74% in 2011, 0.59% in 2012, 0.57% in 2013 and 0.53% in 2014. Estimates of the annual incidence for DUs and FSWs were very low and may not be reliable.

**Conclusions:**

Serial cross-sectional prevalence data from representative samples may be another approach to construct approximate estimates of national HIV incidence among key populations. We observed that the MSM population had the highest incidence for HIV among high-risk groups in China, and we suggest that interventions targeting MSM are urgently needed to curb the growing HIV epidemic.

## Introduction

In 2012, there were approximately 35 (33.2 to 37.2) million people living with HIV and 2.1 (1.9 to 2.4) million new infections globally [1]. HIV incidence is a useful indicator for monitoring the spread of the epidemic and the effectiveness of HIV/AIDS prevention and treatment efforts. However, direct calculation of HIV incidence from epidemiological surveys has proven to be a difficult methodological issue.

Although considered the most traditional way to measure HIV incidence, prospective cohort studies are expensive, time consuming and not typically representative of nationwide trends. Since the 1990s, laboratory-based incidence assays have been developed and introduced into cross-sectional surveys [2–5], and improvements in methods and algorithms have yielded more reliable estimates of new HIV infections [6,7]. However, conducting representative sampling and complicated algorithmic approaches to estimate HIV incidence at a national level is prohibitive for countries with highly concentrated HIV epidemics, such as China [8,9]. UNAIDS has also recommended mathematical models for estimating national HIV incidence rates, such as the Estimation and Projection Package (EPP) [10–12], Spectrum [13–15], Asian Epidemic Model (AEM) [16] and Modes of Transmission (MOT) model [17]. However, the application of these models is limited by the difficulty of defining subpopulations and sub-epidemics in countries with concentrated epidemics and a lack of valid parameter estimates for many resource-limited subnational areas.

In a relatively closed population, HIV prevalence is affected by both incidence and case fatality. Because cross-sectional surveys can be used to capture HIV prevalence and HIV-related mortality, methods have been proposed for estimating incidence based on these observed measures. Previous research in sub-Saharan African countries has shown that sufficiently accurate estimates of incidence can be generated from cross-sectional prevalence data on generalized epidemics [18–21].

The HIV epidemic in China is primarily concentrated among high-risk groups. Drug users (DUs) [22], female sex workers (FSWs) [23,24] and men who have sex with men (MSM) [25,26] are the three main subpopulations affected by HIV in China. In order to monitor the HIV epidemic, China established the HIV Sentinel Surveillance System (HSSS) in 1995 to conduct cross-sectional surveys among eight targeted subpopulations designated as key affected populations (KAPs) [22,27]. Following a rapid expansion of the HSSS in 2010 [28], this system is now a nationwide surveillance network, which provides a way to record changes in the annual HIV prevalence among high-risk populations, including DUs, FSWs and MSM. The aim of this paper is to describe trends in HIV prevalence among high-risk populations in China and to estimate incidence using a mathematical model.

## Methods

### HIV Sentinel Surveillance

**Figure 1:**
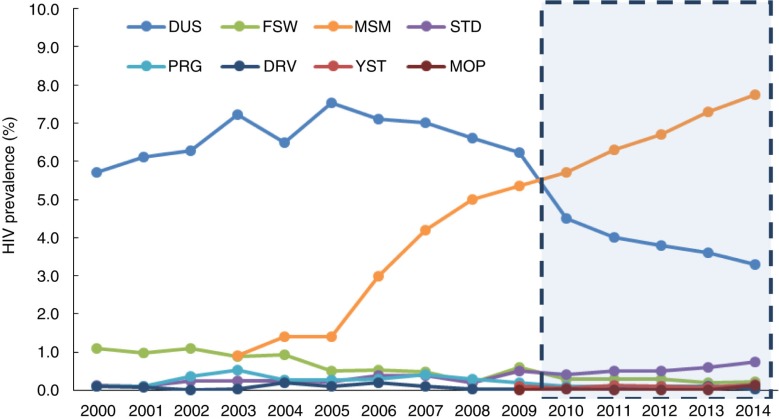
Study period from 2010 to 2014. DUs=drug users, FSW=female sex workers, MSM=men who have sex with men, STD=male patients of sexually transmitted disease clinics, PRG=pregnant women, DRV=long-distance truck drivers, YST=young (college-age) students, MOP=male mobile population (migrant workers).

The introduction and development of the HSSS has been described in other papers [27–30]. The number of sentinel sites has risen from 42 initial sites in 1995 to 101 sites in 2000 and to 600 sites in 2009. In 2010, the HSSS was further scaled up by introducing new sentinel sites in areas where high-risk groups were most concentrated. In total, 1888 sentinel sites were established within 300 cities across 31 provinces. The HSSS targets the following eight KAPs: DUs, MSMs, FSWs, male patients of sexually transmitted disease (STD) clinics, pregnant women, long-distance truck drivers, college students and male migrant workers [28]. This study analysed survey data collected from 2010 to 2014 (Figure 1) at sentinel sites nationwide.

Cross-sectional surveys were conducted among the eight KAPs from April to June each year. The sample size for each surveillance site was 400 participants across the KAPs except for college students. Separate sites were established at colleges and had a target of reaching 800 students. Venue-based sampling in the first year was used to randomly select sites based on location and opening time, and participants were recruited consecutively at venues. Surveys were conducted at the same sentinel sites each year, and the annual target sample size at each site was fixed. After providing informed consent, participants completed an anonymous, structured questionnaire which covered demographic information, HIV-related behaviours and HIV testing history. HIV testing was recommended for all participants. Testing was done by two enzyme-linked immunosorbent assays (ELISA), and participants were not informed of their result unless they requested it by a self-defined encryption code [28].

Participants with two positive ELISA results in the surveillance system were not reported to the national HIV case reporting system until the participants were notified and received western blot confirmatory testing [31]. Per national policy, individuals with a confirmed HIV diagnosis are then registered in the national case reporting system, assigned a unique identification number, followed up for health consults at least every six months and initiated on antiretroviral therapy (ART) if eligible. Patient data, including CD4 count [32], ART regimen [33], HIV/AIDS clinical stage and death, are uploaded to the case reporting system by the follow-up healthcare providers [33]. HIV case fatality was calculated by the proportion of observed deaths in the following year among HIV-positive individuals diagnosed through the cross-sectional surveys and reported to the case reporting system.

The HIV prevalence and interquartile range (IQR) among each target population was calculated at the site and national levels for each year from 2010 to 2014. Chi-square tests were performed to assess the differences among subgroups within KAPs. Trends in HIV prevalence were assessed by the Cochran-Armitage test using SAS software version 9.3 (SAS Institute Inc., Cary, NC, USA).

### Estimation of HIV incidence

To estimate HIV incidence, the DU HIV prevalence was adjusted by the distribution of DU types: “traditional” DU, club DU and user of both traditional and club drugs. Traditional drugs are defined in sentinel surveillance guidelines as heroin, opium, opiate analgesics, cocaine and marijuana. Heroin use through injection accounts for the majority of drug use in China. Club drugs include methamphetamine, ketamine, 3,4-methylenedioxy-N-methylamphetamine (MDMA) and “Magu” pills, which are a mixture of methamphetamine and caffeine [22]. The HIV prevalence among MSM and male STD patients were adjusted by cities, and the FSW HIV prevalence was adjusted by venue (low-fee or other).

We estimated incidence utilizing serial prevalence estimates and HIV survival based on the method described by Brookmeyer and Konikoff [34]. Generally, the method proposes that changes in HIV prevalence can be related to incidence and survival through a balancing equation. Suppose two cross-sectional surveys for HIV prevalence were conducted at times *t*_*1*_ and *t*_*2*_ in a key population. Let *P*_*1*_ indicate the HIV prevalence at time *t*_*1*_ (i.e. the proportion of living persons who are HIV-infected), and *Q*_*1*_=1-*P*_*1*_ the proportion of living persons at time *t*_*1*_ that are not infected but at risk of HIV infection. Similarly, *P*_*2*_ is the HIV prevalence at *t*_*2*._ The relative survival (*R*) of the HIV-infected population (relative to the whole targeted population) is the ratio of the survival probability that HIV-infected persons survive the period δ (where δ=*t*_*2*_−*t*_*1*_) divided by the corresponding probability for the entire population. It is shown^30^ that incidence can be estimated from the equation

1 $$I=(P_{2}-P_{1}R)/Q_{1}\delta$$

If *P*_2_<*P*_1_*R*, then we set *I* to a low value, such as <0.001, indicating a low estimate of new HIV infections. Because of low mortality among the general HIV-negative population over 1 year, we assumed that the survival of the entire population is approximately 1; thus, relative survival *R* is equal to 1 minus the case fatality.

Studies have found that average duration of engagement as a FSW in China is approximately 3 to 5 years, which means that one-fifth to one-third of FSWs will leave this population each year (i.e. turnover) [35]. We assumed that turnover occurs randomly irrespective of HIV status, new entering FSWs are HIV-negative, and the total FSW population size is constant over years. Thus, among FSWs, the relative survival (*R)* is the combination parameter of turnover (*T*_*i*_) and case fatality (*F*_*i*_). The relative survival (*R*) of HIV among FSWs can be expressed as

2 $$R=(1-T_{i})\times (1-F_{i})$$

### Ethics

This study was reviewed and approved by the Institutional Review Board of the National Centre for AIDS/STD Prevention and Control, Chinese Centre for Disease Control and Prevention. All participants provided written informed consent before participating in the surveys. Study participants were reimbursed 50 RMB (~8 USD) for the cost of transportation.

## Results

### HIV prevalence

Cross-sectional surveys were conducted annually from 2010 to 2014. The sample sizes of the surveys were 731,916 in 2010, 757,543 in 2011, 772,681 in 2012, 777,084 in 2013 and 749,116 in 2014. The crude HIV prevalence estimates for all participants were 1.10% for 2010, 1.07% for 2011, 1.12% for 2012, 1.13% for 2013 and 1.17% for 2014 (Table 1).

**Table 1:** HIV prevalence among key affected populations in serial cross-sectional surveys, 2010 to 2014

	2010			2011			2012			2013			2014		
					
	Sample size	Prevalence (%) (IQR)	*P’* (%)	Sample size	Prevalence (%) (IQR)	*P’* (%)	Sample size	Prevalence (%) (IQR)	*P’* (%)	Sample size	Prevalence (%) (IQR)	*P’* (%)	Sample size	Prevalence (%) (IQR)	*P’* (%)
Drug users	108,636	4.50 (0.3 to 4.7)	4.50	111,271	4.02 (0 to 5.5)	4.19	116,216	3.87 (0 to 3.5)	4.21	117,576	3.61 (0 to 3.8)	4.20	116,654	3.31 (0 to 3.3)	4.08
IDU	61,215	6.95 (0.3 to 8.9)	NA	62,076	6.43 (0 to 1.65)	NA	62,923	6.31 (1.1 to 6.1)	NA	58,066	6.33 (1.1 to 5.9)	NA	52,787	6.00 (0.9 to 5.8)	NA
Female sex workers	198,083	0.24 (0 to 0.2)	0.24	203,814	0.23 (0 to 0.2)	0.23	206,893	0.24 (0 to 0.3)	0.24	208,264	0.24 (0 to 0.2)	0.19	185,798	0.17 (0 to 0.2)	0.16
Men who have sex with men	34,009	5.73 (2.0 to 9.6)	5.73	37,094	6.32 (2.8 to 9.3)	6.35	39,949	6.69 (3.3 to 10.3)	6.81	42,582	7.30 (3.5 to 10.7)	7.29	42,951	7.75 (3.5 to 11.8)	7.72
Male STD patients	137,094	0.43 (0 to 0.5)	0.43	145,135	0.45 (0 to 0.5)	0.45	147,506	0.52 (0 to 0.5)	0.52	147,772	0.56 (0 to 0.5)	0.55	143,906	0.74 (0 to 1.0)	0.73
Pregnant women	148,673	0.06 (0 to 0)	NA	150,884	0.06 (0 to 0)	NA	151,770	0.09 (0 to 0)	NA	150,938	0.08 (0 to 0)	NA	150,784	0.05 (0–0)	NA
College students	50,452	0.02 (0 to 0)	NA	52,178	0.01 (0 to 0)	NA	52,200	0.02 (0 to 0)	NA	52,248	0.00 (0 to 0)	NA	52,387	0.04 (0 to 0)	NA
Long-distance drivers	23,369	0.05 (0 to 0)	NA	23,263	0.05 (0 to 0)	NA	23,145	0.03 (0 to 0)	NA	22,958	0.04 (0 to 0)	NA	21,730	0.04 (0 to 0)	NA
Male migrant workers	31,600	0.06 (0 to 0)	NA	33,904	0.12 (0 to 0.2)	NA	35,002	0.10 (0 to 0.2)	NA	34,746	0.07 (0 to 0)	NA	34,906	0.14 (0 to 0.2)	NA
Total	731,916	1.10	NA	757,543	1.07	NA	772,681	1.12	NA	777,084	1.13	NA	749,116	1.17	NA

STD=sexually transmitted disease; IDU=injecting drug user among all drug users; IQR=interquartile range; P’=adjusted prevalence; NA=not applicable

**Figure 2:**
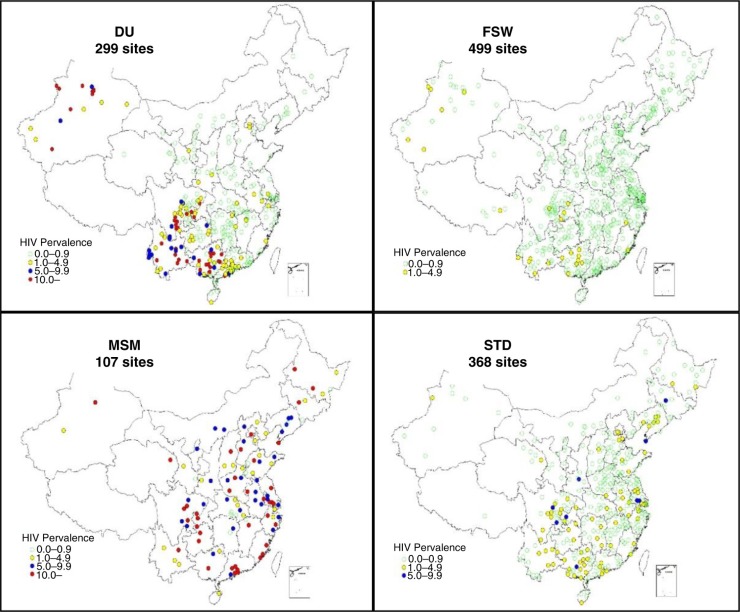
Geographical distribution of HIV prevalence among drug users (DU), female sex workers (FSW), men who have sex with men (MSM) and male patients of sexually transmitted disease clinics (STD) in 2014.

The geographical distribution of HIV prevalence among DUs, FSWs, MSM and male patients of STD clinics in 2014 is presented in Figure 2. Among DU participants from 299 survey sites in 2014, 168 sites reported an HIV prevalence <1%, 70 sites reported between 1 and 5%, 61 sites reported between 5 and 10% and 39 sites reported >10%. Almost all sites with DU HIV prevalence higher than 5% were in the provinces of Guangxi, Sichuan, Yunnan and Xinjiang. Stratified by drug types, DUs who used only traditional drugs had higher HIV prevalence (4.86%; 3440/70,724) than club DUs (0.47%; 168/35,811) and concurrent users of both traditional and club drugs (2.43%; 3829/9099; *p*<0.0001).

In 2014, FSW were surveyed at 499 sites. The HIV prevalence at 473 sites was less than 1%, and the remaining 26 sites had a prevalence between 1 and 5%. FSWs who worked at low-fee venues had a higher HIV prevalence (0.36%; 211/58,976) than other FSWs (0.08%; 98/126,046; *p*<0.0001).

All sites that surveyed MSM, except for two, identified HIV-infected individuals. MSM in large cities experienced a higher HIV prevalence (10.73%; 1534/14,297) than MSM in other cities (6.26%; 1793/28,654; *p*<0.0001).

Among male patients of STD clinics at 368 sites, 274 sites reported <1% HIV prevalence, 84 sites reported between 1 and 5%, and 10 sites reported higher than 5%. Participants from large cities had a higher prevalence (1.92%; 368/19,187) than other cities (0.56%; 694/124,719; *p*<0.0001).

### Trends in HIV prevalence and case fatality

From 2010 to 2014, HIV prevalence among DUs decreased from 4.50 to 3.31% while the prevalence among MSM increased from 5.73 to 7.75%. HIV prevalence among FSWs remained low with slight fluctuations between 0.17 and 0.24%. Among male patients of STD clinics, the prevalence increased from 0.43 to 0.74% (Table 1). The Cochran-Armitage test for trend showed that the changes in HIV prevalence among DUs (*p*<0.0001), FSWs (*p*<0.0001), MSM (*p*<0.0001) and male patients of STD clinics (*p*<0.0001) were significant.

From 2011 to 2013, the respective annual case fatality (Table 2) among the surveyed DUs was 1.6% (*N*=3161), 1.7% (*N*=3321) and 2.4% *(N*=2996); and among FSWs, the annual case fatality was 0.9% (*N*=212), 0.5% (*N*=384) and 0.3% *(N*=293). Among MSM, the case fatality was 1.4% (*N*=1025), 0.9% (*N*=1610) and 1.3% *(N*=1502); and among male patients of STD clinics, the case fatality was 5.3% *(N*=243), 2.5% (*N*=398) and 2.1% (*N*=375).

**Table 2:** Case fatality among HIV positive KAPs detected during cross-sectional surveys from 2011 to 2013

	2011^a^		2012^a^		2013^a^	
			
KAP	WB confirmed	One year fatality rate* (%)	WB confirmed	One year fatality rate (%)	WB confirmed	One year fatality rate (%)
DUs	3161	1.6	3321	1.7	2996	2.4
FSW	212	0.9	384	0.5	293	0.3
MSM	1025	1.4	1610	0.9	1502	1.3
STD	243	5.3	398	2.5	375	2.1

a Years of cross-sectional survey; KAP=key affected population; WB=western blot; DUs=drug users; FSW=female sex workers; MSM=men who have sex with men.

### HIV incidence

**Figure 3:**
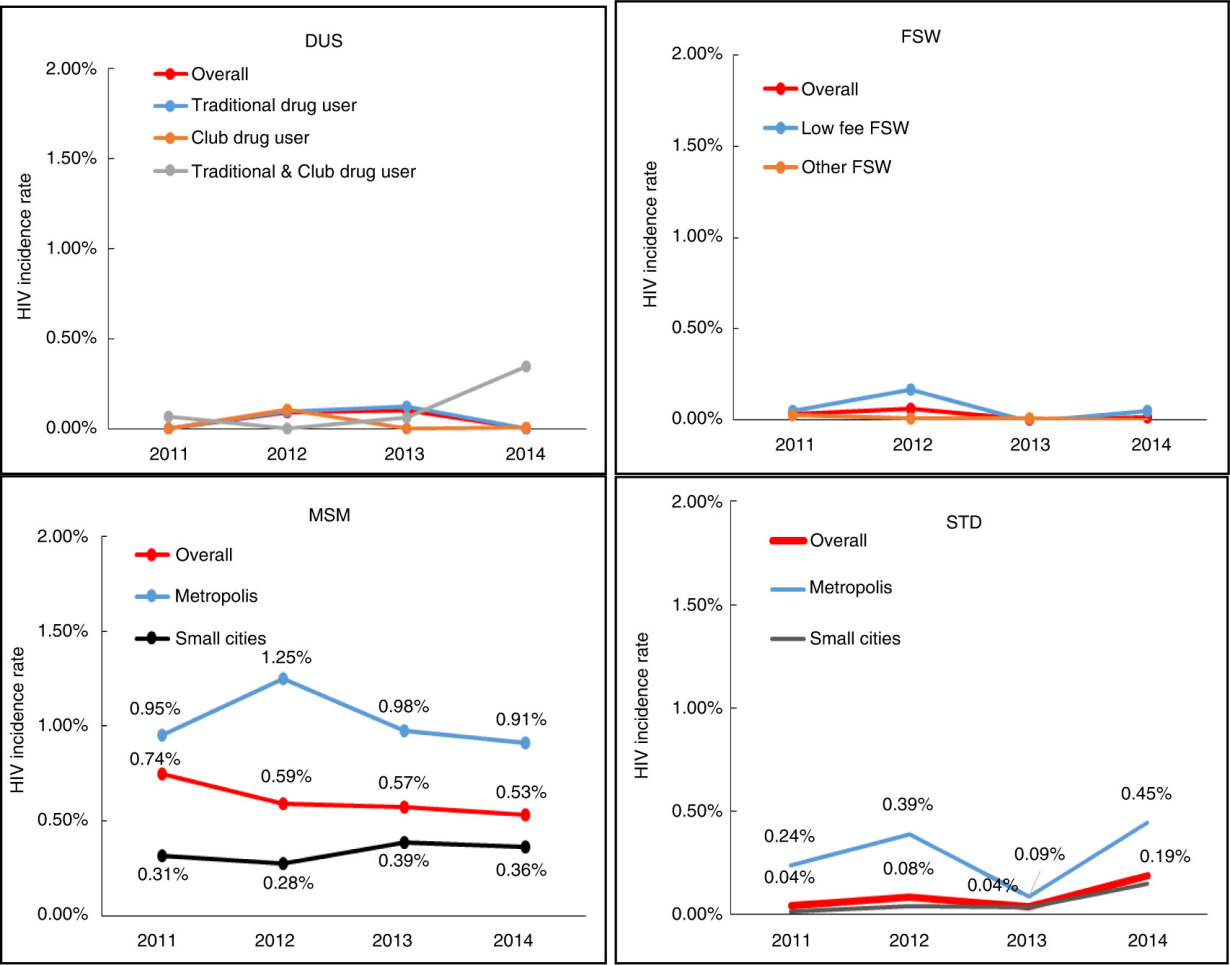
HIV incidence among key populations from 2011 to 2014. DUs=drug users, FSW=female sex workers, MSM=men who have sex with men, STD=male patients of sexually transmitted disease clinics.

Based on Equations (1 and 2), we estimated the HIV incidence from 2011 to 2014 among DUs, FSWs, MSM and male patients of STD clinics (Figure 3). An adjusted HIV prevalence (*P’*) was used during HIV incidence estimation. We calculated that the DU HIV incidence estimates were <0.001% in 2011, 0.09% in 2012, 0.10% in 2013 and <0.001% in 2014. FSWs had an estimated incidence of 0.03% in 2011, 0.06% in 2012, <0.001% in 2013 and 0.01% in 2014. Among MSM, the incidence estimates were 0.74% in 2011, 0.59% in 2012, 0.57% in 2013 and 0.53% in 2014. Among male patients of STD clinics, the incidence was 0.04% in 2011, 0.08% in 2012, 0.04% in 2013 and 0.19% in 2014. Our data showed that the HIV incidence was notably high among MSM in large cities at about 0.95% in 2011, 1.25% in 2012, 0.98% in 2013 and 0.91% in 2014. In comparison, the incidence among MSM in smaller cities was 0.31% in 2011, 0.28% in 2012, 0.39% in 2013 and 0.36% in 2014.

## Discussion

Measuring incidence is a valuable method for describing and monitoring changes in the HIV epidemic. Using data from a series of annual cross-sectional surveys in China from 2010 to 2014, we examined changes in HIV prevalence across key high-risk groups. We produced estimates of HIV incidence in the context of a centralized epidemic based on the relationship among prevalence, case fatality and incidence [34].

In 2010, the Chinese national HSSS was widely scaled up across the country to better characterize and monitor the HIV epidemic [28]. All sentinel sites conduct annual HIV prevalence surveys, targeting key populations at high risk for HIV acquisition. We found that the MSM population, especially MSM living in large cities, experienced the highest HIV prevalence and incidence from 2010 to 2014 among all KAPs. MSM in China are at exceptionally high risk for HIV with the prevalence rising from 5.73% in 2010 to 7.75% in 2014. These estimates are an unsurprising increase from the findings reported in our previous study of MSM in 61 cities conducted from February 2008 to September 2009 [25], which found a prevalence of 4.9% (95% confidence interval: 4.7 to 5.1%).

During the same time period, HIV prevalence among DUs followed a decreasing trend. Injection of opioids has historically been the main driver of the HIV epidemic in China, but over the last decade, sexual transmission of HIV has become the most common route of transmission. In our 2014 surveillance survey, “traditional” DUs had a nearly 10-fold higher HIV prevalence (4.86%) than club DUs (0.47%). Drug use trends are shifting from predominantly opioid use to increasingly common use of club drugs such as methamphetamine, MDMA (“ecstasy”) and ketamine. A previous study of surveillance survey data reported a rapid increase in club drug use from 1.3% of DUs in 2004 to 24.4% in 2011 [22]. Data from the government have shown that 45% of registered DUs used club drugs in China in 2014 [36]. The change in drug use patterns presents a serious challenge for public health programmes and has unclear implications for HIV control.

We found a low and decreasing HIV prevalence for FSWs in China from 2010 to 2014. The decreasing trend among FSWs may be because of the ongoing condom use campaign in place since 2003 [37] and the turnover effect in this population, which is approximately one-fifth to one-third of FSWs [23].

For male patients of STD clinics, HIV prevalence increased between 2010 and 2014. We believe there are several possible mechanisms behind this trend. Increased engagement in the Chinese sex industry, which has expanded rapidly over the past three decades, is thought to be linked to a recent sharp rise in syphilis and other STDs [38]. The increase in HIV prevalence among STD patients might also be attributable to increased rates of diagnosis and case-reporting practices of STDs [39] or changes in healthcare-seeking behaviour among MSM and clients of FSWs. We also found that STD patients in larger cities were more likely to be HIV-positive, but it is unknown whether this reflects a true rise in disease or better access to healthcare in larger cities.

In comparison to prevalence, incidence is a more direct and sensitive indicator of epidemic dynamics. We estimated that the MSM population had the highest overall incidence during our study period among all high-risk groups, but encouragingly, our incidence estimates show a decline from 0.74% in 2011 to 0.53% in 2014. When stratified by size of cities, the HIV incidence among MSM living in large cities is much higher than among MSM in smaller cities but has been declining since 2012. In contrast, the incidence in smaller cities is lower overall but has increased from 0.28% in 2012 to 0.39% in 2013 and to 0.36% in 2014. According to a national report characterizing the HIV epidemic in China, HIV-positive MSM often choose to live in larger cities [40]. To address the HIV epidemic among MSM, the Chinese government has been expanding targeted campaigns to encourage HIV testing and to increase engagement in care for urban MSM populations since 2012. Our findings also highlight the urgent need for a comprehensive response against the HIV epidemic among MSM in smaller cities.

Our estimates of MSM HIV incidence are much lower than previously published incidence rates measured through BED (HIV-1 subtypes B, E, and D) capture enzyme immunoassay (BED-CEIA) testing and prospective cohort studies in many metropolitan areas, including Beijing, Chengdu and Shenyang [41,42], but similar to an systematic review estimate of HIV incidence among MSM of 0.98/100 person-years (95% confidence interval: 0.70 to 1.25/100 person-years), as calculated by Spectrum/EPP software [43]. BED-CEIA methods tend to overestimate incidence when using cross-sectional survey data compared with cohort study data [44,45] and are subject to the sampling bias in a single cross-sectional survey. Spectrum/EPP methods and our methods are based on similar cross-sectional surveys, which may also be biased because of disproportionate sampling of subgroups within populations. We attempted to limit the impact of selection bias, allowing us to produce reasonable estimates of HIV incidence, by using survey data from multiple years across many sites and adjusting for subgroups within the key populations.

Our HIV incidence estimates suggest that there has been substantial progress in controlling the spread of HIV among DUs. The incidence among DUs was less than 0.1% from 2011 to 2014. Nationwide harm reduction programmes in place since 2006 [46] and the recent shift to increased club drug use [22] might contribute to the low HIV incidence among DUs. It is notable that, among those who use both traditional and club drugs, HIV incidence increased from <0.001 in 2012 to 0.06% in 2013 and 0.35% in 2014. Club drug use has an unclear relationship with HIV risk in China. Several studies have suggested that a rise in club drug use led to a subsequent increase in HIV prevalence through high-risk sexual behaviour [47,48]. Additional research studies and public health initiatives are needed to understand and combat the HIV risk associated with club drug use.

The low HIV incidence among FSWs and male patients of STD clinics are not surprising. However, because of the total population size of FSWs and their clients in China, even a very low HIV incidence among FSWs can generate a large number of new HIV infections. The latest surveillance data showed that among the 103,501 newly-diagnosed HIV/AIDS patients in 2014, about two-thirds (66.4%) self-reported as having been infected through heterosexual contact [49].

Most past studies that estimated HIV incidence from cross-sectional survey data have been conducted in generalized epidemic settings in sub-Saharan Africa, where surveys were household-based [21]. Because of the extremely low HIV prevalence among the general population in China (estimated to be less than 0.06% in 2011) [40], representative household-based surveys are not generally feasible in China. Compared to other studies in sub-Saharan Africa, our study had several limitations. First, valid estimation of incidence assumes that the cross-sectional surveys are representative of the target population and not subject to significant selection bias. Although we adjusted the HIV prevalence (*P*_*i*_) estimates by target population subgroups (e.g. low-fee venue-based FSWs compared with FSWs who work in other settings), during incidence calculations, we are not able to account for all selection bias. Another potential source of bias is loss to follow-up of participants who screen HIV-positive but fail to obtain confirmation testing. These individuals are not counted among the HIV-infected population in our analysis, but we do not believe that loss to follow-up varied across years and would have a minimal impact on our findings. Second, because of the very low HIV prevalence among FSWs and male STD patients, we found extremely low incidence over our study period, which may be unreliable. Of note, we assumed that HIV-infected and HIV-uninfected FSW leave the FSW population at similar rates, as we could not find published research on this topic. Third, the migration of KAPs may be a source of bias in the HIV prevalence trends. In this study, we attempted to reduce the impact of migration on HIV incidence estimates by presenting HIV prevalence at the national level based on data collected annually at same sentinel sites; target sample sizes per survey site remained consistent across study years.

## Conclusions

In the context of concentrated epidemics, valid HIV incidence estimates can be difficult to ascertain. From serial HIV prevalence and case fatality data collected across China, we used a mathematical model to estimate HIV incidence among key high-risk populations, focussing on DUs, FSW, MSM and male STD patients. We found large differences in prevalence and incidence among the target populations, which has implications for prioritizing and designing future HIV/AIDS prevention efforts. In particular, our results highlight the high HIV risk experienced by the MSM population. Future estimates of HIV incidence can be improved by increasing the representativeness of the surveillance surveys.
